# Supplementary material for: From sequence to enzyme mechanism using multi-label machine learning
Source: BMC Bioinformatics. 2014 May 19;15:150. doi: 10.1186/1471-2105-15-150 (PMC4229970; doi:10.1186/1471-2105-15-150)
Supplement: Additional file 2 — Java code of ml2db. Additional file ml2db_code.tar.gz contains the Java source code to run the multi-label machine learning experiments and save the results to database. The code’s Javadoc is included. [file 1471-2105-15-150-S2.zip › additional file 2/ml2db/ml2db/doc/mulan/data/package-use.html]

Uses of Package mulan.data


JavaScript is disabled on your browser.


- Overview
- Package
- Class
- Use
- Tree
- Deprecated
- Index
- Help

- Prev
- Next

- Frames
- No Frames

- All Classes

# Uses of Package mulan.data

- Packages that use mulan.data

  | Package | Description |
  | --- | --- |
  |  |  |
  | --- | --- |
  | mulan.classifier |  |
  | mulan.classifier.meta |  |
  | mulan.classifier.meta.thresholding |  |
  | mulan.classifier.neural |  |
  | mulan.classifier.transformation |  |
  | mulan.data |  |
  | mulan.dimensionalityReduction |  |
  | mulan.evaluation |  |
  | mulan.evaluation.loss |  |
  | mulan.transformations |  |
  | mulan.transformations.multiclass |  |
- Classes in mulan.data used by mulan.classifier

  Class and Description

  MultiLabelInstances

  Implements multi-label instances data set.
- Classes in mulan.data used by mulan.classifier.meta

  Class and Description

  InvalidDataFormatException

  The exception is thrown when format of the data is not valid.

  LabelClustering

  An interface for various label clustering algorithms.

  LabelPairsDependenceIdentifier

  An interface for various types of dependency identification between pairs of labels.

  LabelsMetaData

  Represents meta data about label attributes and their structure.

  MultiLabelInstances

  Implements multi-label instances data set.
- Classes in mulan.data used by mulan.classifier.meta.thresholding

  Class and Description

  MultiLabelInstances

  Implements multi-label instances data set.
- Classes in mulan.data used by mulan.classifier.neural

  Class and Description

  MultiLabelInstances

  Implements multi-label instances data set.
- Classes in mulan.data used by mulan.classifier.transformation

  Class and Description

  MultiLabelInstances

  Implements multi-label instances data set.
- Classes in mulan.data used by mulan.data

  Class and Description

  InvalidDataFormatException

  The exception is thrown when format of the data is not valid.

  LabelClustering

  An interface for various label clustering algorithms.

  LabelNode

  Represents a label attribute as a node in the labels hierarchy.

  LabelPairsDependenceIdentifier

  An interface for various types of dependency identification between pairs of labels.

  LabelsBuilderException

  Exception is raised by `LabelsBuilder` to indicate an error when creating
  `LabelsMetaDataImpl` instance form specified source.

  LabelSet

  Class that handles labelsets

  LabelsMetaData

  Represents meta data about label attributes and their structure.

  LabelsPair

  Class for handling label pairs along with their 'dependence' scores.

  MultiLabelInstances

  Implements multi-label instances data set.

  Stratification

  Interface for multi-label stratification methods
- Classes in mulan.data used by mulan.dimensionalityReduction

  Class and Description

  MultiLabelInstances

  Implements multi-label instances data set.
- Classes in mulan.data used by mulan.evaluation

  Class and Description

  MultiLabelInstances

  Implements multi-label instances data set.
- Classes in mulan.data used by mulan.evaluation.loss

  Class and Description

  MultiLabelInstances

  Implements multi-label instances data set.
- Classes in mulan.data used by mulan.transformations

  Class and Description

  MultiLabelInstances

  Implements multi-label instances data set.
- Classes in mulan.data used by mulan.transformations.multiclass

  Class and Description

  MultiLabelInstances

  Implements multi-label instances data set.

- Overview
- Package
- Class
- Use
- Tree
- Deprecated
- Index
- Help

- Prev
- Next

- Frames
- No Frames

- All Classes
